# Secure Cluster Head Sensor Elections Using Signal Strength Estimation and Ordered Transmissions

**DOI:** 10.3390/s90604709

**Published:** 2009-06-16

**Authors:** Gicheol Wang, Gihwan Cho

**Affiliations:** 1 Department of Computing and Networking Resources, 335 Gwahangno, Yuseong-gu, KISTI / Daejeon, 305-806, Korea; 2 CAIIT, Div. of Electronics and Information Engineering, Chonbuk Univ. / Jeonju, 561-756, Korea; E-Mail: ghcho@chonbuk.ac.kr

**Keywords:** secure cluster head election, sensor networks, signal strength estimation, ordered transmissions

## Abstract

In clustered sensor networks, electing CHs (Cluster Heads) in a secure manner is very important because they collect data from sensors and send the aggregated data to the sink. If a compromised node is elected as a CH, it can illegally acquire data from all the members and even send forged data to the sink. Nevertheless, most of the existing CH election schemes have not treated the problem of the secure CH election. Recently, random value based protocols have been proposed to resolve the secure CH election problem. However, these schemes cannot prevent an attacker from suppressing its contribution for the change of CH election result and from selectively forwarding its contribution for the disagreement of CH election result. In this paper, we propose a modified random value scheme to prevent these disturbances. Our scheme dynamically adjusts the forwarding order of contributions and discards a received contribution when its signal strength is lower than the specified level to prevent these malicious actions. The simulation results have shown that our scheme effectively prevents attackers from changing and splitting an agreement of CH election result. Also, they have shown that our scheme is relatively energy-efficient than other schemes.

## 1. Introduction

Currently cluster structures are frequently employed in wireless sensor networks. These cluster structures enable the energy conservation in sensors [1,2], load balancing [3], distributed key management [4,5], and so on. Generally, transforming a network into a cluster structure is achieved by combining some adjacent sensors into a group and electing a group leader within the group. A group and the leader are called a cluster and a Cluster Head (CH), respectively. In the clustered sensor network, the compromise of CHs is more threatening than that of member sensors, and CHs are also located in the unprotected environment like member sensors. Because CHs are the data collection points, smart attackers may compromise the network by targeting the CHs rather than the other sensors. This is because by compromising all CHs they can gain control of the whole network. A suitable example of the assumed threat model is a military surveillance network. In this network, sensors detect the movement and invasion of enemy troops, and then notify headquarters of the threats. Compromised sensors still obtain the movement or invasion information, but the attackers can forge the information to hide the movement or invasion from headquarters. Then they send the forged information to the sink indicating that there is no suspicious activity. When all of the CHs are compromised, the control of the whole network is given to the enemies and their movement and invasion can go completely undetected. In this case, the invasion of the enemies is completely hidden from the headquarters.

To elect a CH, existing CH election schemes make sensors exchange a criterion such as ID or degree or low mobility or residual energy. Then, they compare the criterion among neighbors [1,2,6-9], and elect CH role nodes by choosing a node with a highest criterion among all its neighbors. A CH role node declares itself as a CH with a broadcast message, and the receivers of the message respond to the CH with a unicast message, and the CH and the responders thus form a cluster. The primary problem of the existing CH election schemes is that legitimate nodes cannot prevent a malicious node from fabricating its criterion and transmitting the fabricated criterion. This gives a malicious node a good chance of becoming a CH.

To resolve this problem, a random value based scheme, called SANE (Secure Aggregator Node Election) [10], was proposed recently. In this scheme, because a CH is elected in a random manner, a naive attacker can neither easily influence the CH election result nor know which node can become a CH in the election. However, an intelligent attacker can manipulate the CH election result as well as generate some redundant CHs. This misbehavior partitions the clusters and might even make a compromised node a CH.

In this paper, we propose a CH election scheme which is resilient to this misbehavior. First, our scheme settles the broadcast order of contributions for random value agreement and forces all sensors to follow the order. If a sensor keeps violating this order, this sensor is considered as a malicious node which is trying to manipulate the CH election result and it is evicted from the contributor list. An attacker may reduce the power level of a contribution message to make receivers have a different set of contributions. It increases the number of CHs in the network and reduces the size of clusters. As a result, energy consumption of sensors increases due to frequent transmission of sensor readings. To prevent this misbehavior, all receivers of a contribution measure the signal strength power of the contribution and infer the approximately reachable distance of the contribution. That is, the receivers discard the contribution whose power level is too weak to reach all sensors in the cluster.

This paper is organized as follows. Section 2 overviews the related work concerning CH election. In Section 3, we describe the network and threat model. Section 4 deals with the preparations for our CH election scheme, and the details of our CH election scheme are described in Section 5. Section 6 compares our scheme with other schemes through experiments, and Section 7 shows how our scheme satisfied the requirements for CH election. Lastly, Section 8 concludes this paper.

## 2. Related Work

Eschenauer and Gilgor were the first to propose a scheme for establishing a communication key using key pre-distribution [11]. In this scheme, any two neighbor sensors establish a pairwise key using common pre-distributed keys. If they have no common keys, then they establish the pairwise key indirectly through proxy nodes. Here, proxy nodes refer to the sensors that share at least one common key with the two nodes. The problem with this scheme is that any two sensors that share only one common key can establish a pairwise key. Therefore, it is very vulnerable to the compromise of sensors. Chan *et al.* resolved this problem by fixing the minimum number of common keys required for pairwise key establishment to *q* (> 1) [12].

Representative schemes which use weights for CH election are LIDCP (Lowest ID Clustering Protocol) [7] and HCCP (Highest Connectivity Clustering Protocol) [7]. LIDCP elects a lowest ID node in the neighborhood as a CH, while HCCP elects a highest degree node in the neighborhood as a CH. The so-called WCA (Weighted Clustering Algorithm) [6] considers degree, transmission power, mobility, and residual energy as criteria for CH election. These criteria are assigned different weights, according to the relative importance of the criteria in the network application. A final criterion is generated by multiplying each criterion by the corresponding weight and summing them. MOBIC (Lowest Relative Mobility Clustering) [8] presented a scheme which elects a CH by comparing relative mobility in the neighborhood. The relative mobility is estimated by measuring received signal power of two consecutive hello messages. Namely, a node exchanges two consecutive messages with neighbors and measures the difference of received signal power between two messages. These values can be positive values or negative values. Each node can get relative mobility by computing the variance with respect to zero. The prominent problem of above weight based schemes is that a malicious node can broadcast a forged criterion as if it has a highest criterion among neighbors. In that case, it can become a CH.

Heinzelman *et al.* proposed LEACH (Low-Energy Adaptive Clustering Hierarchy), which elects a CH without message exchange. This scheme tried to extend the network lifetime by giving all nodes equal chances to be a CH. In this scheme, each sensor becomes a CH or a member of a CH depending on the computed probability. Therefore, the hop distance between a CH and its members can be further than single hop. In HEED [2], nodes elect a CH using their residual energy and communication cost to their neighbors. That is, the initial probability that each sensor becomes a CH depends on its residual energy. Later, sensors that do not belong to any clusters double this probability, and this procedure is repeated until all sensors are served by at least one CH. If a sensor has to choose one of two or more CHs, it chooses one with a fewer communication cost. VCA [9] presented a CH election scheme which considered local topology information as well as residual energy. First, VCA balances the number and size of clusters by considering residual energy and degree in the election process. Second, sensors which belong to two or more clusters choose a CH concerning the energy distribution. However, above schemes cannot prevent a malicious node from declaring itself as a CH, like the weight based schemes.

Ferreira *et al.* proposed F-LEACH [13] to protect the CH election in LEACH. A sensor declares itself as a CH using common keys shared with the sink, and the sink authenticates the CH declaration using the same keys. Then, the sink securely broadcasts the authenticated CHs using μTESLA [14]. Sensors join only one authenticated CH. However, this scheme cannot authenticate the sensors which join the service of a CH. To resolve this problem, Oliveira *et al.* proposed SecLEACH [15] in which the sink authenticates the CH declaration from sensors and CHs also authenticate the joining sensors. In SecLEACH, sensors are assigned some keys for authentication prior to deployment. However, both F-LEACH and SecLEACH can prevent only external attackers from declaring themselves as CHs. That is, they cannot prevent internal attackers from declaring themselves as CHs and joining other CHs.

Recently, Sirivianos *et al.* proposed a CH election scheme using a random value, called SANE. SANE consists of Merkle's puzzle based scheme, a commitment based scheme, and a seed based scheme. In Merkle's puzzle based scheme, a current CH establishes pairwise keys with its members. Then, a member generates its random value and encrypts it using the pairwise key with the current CH. It sums its encrypted random value with the accumulated sum which is received from other node and delivers the sum to another node. This procedure is repeated until all sensors get the total sum of the encrypted random values. To decrypt this sum, each sensor should know all pairwise keys used for the generation of the sum due to the property of homomorphic encryption transformation [10]. So, the current CH distributes the pairwise keys to all nodes, and all nodes get the real sum of random values using the pairwise keys. They divide the real sum of random values by the number of sensors and get the remainder which indicates the position of CH node in the cluster. Because each sensor stores the IDs of nodes in an ascending order, they can easily reach an agreement on the CH election result. This conversion of an agreed random value to a CH position is also applied to other schemes. In the commitment based scheme, each sensor sends its commitment to other sensors in the peer-to-peer manner. Here, a commitment is an encrypted random value using a shared key and the random value is created by each sensor. Then, each sensor sends the fulfillment value (that is, its random value) to other sensors. Receiving sensors verify the fulfillment values using the shared key and sum them to make an agreed random value. In the seed based scheme, each sensor generates its seed value and distributes it to other sensors in the broadcast manner. This seed value is the initial random value for generation of sum of random values. Every CH election round, each sensor broadcasts its availability. This availability is a kind of fulfillment values, and sensors receiving the availability keep the list of the senders. That is, all sensors make a sum of random values using the seed values of the senders and the number of CH election round. Merkle's puzzle based scheme causes a lot of overhead due to the pairwise key establishment, generation of sum of encrypted random values, and the key distribution. The commitment based scheme and the seed based scheme are vulnerable to transmission suppression and selective transmission of fulfillment values. The transmission suppression of fulfillment values causes changes of CH election result. Besides, selective transmission of fulfillment value causes the partition of clusters by separating one agreement of CH election into two or more agreements.

## 3. Network and Threat Model

### 3.1. Network Model

In this paper, sensors are deployed by a helicopter or an airplane without human intervention and they reside in quasi-stationary state during network operation. To produce a cluster structure, one of CH election schemes in Section 2 can be used. However, they are very vulnerable to illegal CH declaration of disqualified nodes as described in Section 2. To debilitate these attackers, a CH election result should be determined in a random manner like SANE [10]. In SANE, sensors are deployed in their pre-assigned sectors, and the CH election result in a sector is independent from other sectors. However, because the network that we assumed has no pre-assigned sectors, sensors need to invoke a CH election scheme to generate sectors after the deployment. We exploit a weight based CH election scheme to complete the sector formation in a short time. Sensors should exchange a criterion (for instance, ID, degree, residual energy and so on) with their neighbors. Then, they elect a local manager, which is called sector manager, by comparing the criteria between neighbors, like weight based election schemes. After this initial cluster formation, the network is divided into multiple sectors and each sector has its own sector manager which plays a role of a helper node for intra-sector key establishments between sensors. After the intra-sector keys are established, the sector manager is treated like a normal sensor in its sector and does not have any special duties. Details related with the sector determination are dealt in Section 4.2. Then, sensors in each sector establish pairwise keys with other sensors for intra-cluster communication. This is because a CH is elected randomly in a sector, and the CH and its members should exchange data directly. For this purpose, each sensor is randomly assigned some keys from the sink with a key pool, and it can know the IDs of the assigned keys of other sensors. If any two sensors have the same assigned key(s), they can establish a pairwise key using them. We deal with the details about pairwise key establishment in Section 4.1 and Section 4.3. Here, we add two reasonable assumptions to the sector determination and the pairwise key establishment. First, all sensors are trustworthy at network boot-up time and this trustworthiness lasts during these two steps. Second, attackers cannot compromise any legitimate sensors during these two steps. Note that these two steps are invoked only one time after the deployment of sensors and these two steps are completed in a very short time. After the pairwise key establishment, sensors in a sector elects one node as their CH. Note that the messages exchanged for CH election are not propagated to other sectors. This is because all sectors exploit different spreading code in their sector as described in Section 4.2.

**Figure 1.**
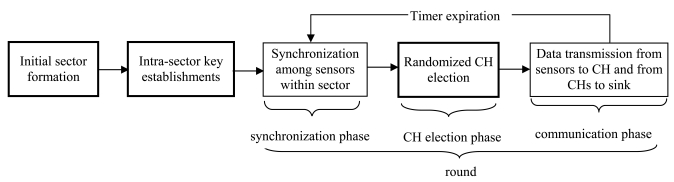
Network operation of clustered sensor networks.

Network operation is divided into rounds, and each round consists of synchronization phase, CH election phase, and communication phase. In the synchronization phase, each sensor synchronizes its clock with other members of the sector to start the CH election at the same time. In the CH election phase, sensors in each sector elect one node as CH and join the service of the CH. To avoid the interference in a sector, elected CHs generate a TDMA schedule and broadcast it. In the communication phase, each sensor transmits its sensed data to its CH according to the schedule. The CH aggregates the received data from sensors and sends the aggregated data to the sink. Each sensor sends its data in its allowed time slots and remains in sleep state in other slots. To save the energy consumption for frequent CH election, the communication phase consists of multiple TDMA frames. CHs employ two levels of power and directly communicate with the sink through a high power level. Figure 1 shows the network operation of clustered sensor networks and the thick rectangles are functions covered in this paper.

### 3.2. Threat Model

Attackers compromise legitimate nodes and participate in the CH election of the legitimate nodes. In this paper, we assume that random value based schemes [10] are employed for the CH election. As described in Section 2, the commitment based scheme and the seed based scheme fall into the category. Our scheme takes advantage of both schemes and adds new mechanisms to mitigate their disadvantages, so our scheme also belongs to this category. The primary goal of attackers is to elect the compromised nodes as CHs. To achieve the goal, attackers need to prevent legitimate nodes from being elected as a CH by changing the CH election result. The subsidiary goal of attackers is to split a cluster into several clusters to facilitate the energy exhaustion of sensors significantly. As the number of clusters in a network increases, the size of a cluster (the number of members in the cluster) decreases. As a result, transmission schedule of a cluster is shortened and sensors in the cluster should transmit its reading more frequently. It shortens the lifetime of sensors and thereby reduces the network lifetime. To actualize above attacks, attackers employ the following methods.

In a random value based scheme, an attacker cannot anticipate which node will be a CH in the round. However, an attacker which has already received fulfillment values of all other sensors can know that. So, an attacker which distributes its fulfillment value last can modify the CH election result by suppressing its fulfillment value distribution. If an attacker keeps modifying the CH election results, a compromised node can become a CH at some point. Especially, as the number of compromised nodes increases, the first goal can be achieved easily. If an attacker transmits its fulfillment value with a low transmission power, the value is delivered to only some part of nodes. In this case, nodes which do not receive the fulfillment value have a different random value from other receivers. So, these nodes may elect a different node as CH. This splits a cluster into several ones.

## 4. Sector Formation and Pairwise Key Establishments

### 4.1. Exchange of ID and Neighbor List

In a network which is divided into sectors, sensors communicate with their CH directly. Note that a CH is selected randomly in a sector. Therefore, the maximum hop distance between a sensor and its CH is two. This means that each sensor should know the IDs of at most two hop sensors to complete pairwise key establishments in its sector. This is because each sensor can know their assigned keys if it recognizes their IDs. For this purpose, each sensor exchanges its ID and neighbor list consecutively at network boot-up time. Through these exchanges, each sensor recognizes other sensors which share common assigned keys within at most two hops, and performs pairwise key establishments in its sector via their help. Hereafter, these sensors sharing common assigned keys are referred as helper nodes. If a sensor node has more helper nodes, it can easily establish pairwise keys with other members in its sector without causing any extra overhead. If we want a sensor to have many helper nodes, we have to assign the same keys to many other nodes. We deal with how this key assignment probability affects the intra-sector key establishments in Section 4.3.

### 4.2. Sector Formation

After exchanging the ID and the neighbor list, sensors invoke a CH election scheme to determine their sector. For instance, if HCCP [7] is used for the sector formation, a sensor compares its degree (number of neighbors) with its neighbors. If it is a highest degree node among neighbors, it becomes a sector manager and broadcasts the manager declaration message to its neighbors. The neighbors become members of the sector and send a join message to the sector manager. Otherwise, it waits for a higher degree node to declare as a sector manager or join as a member to a different sector. Once a sensor joins a sector, it never joins other sectors even if it receives a manager declaration message from a different sector manager. Generally, a CH election scheme creates some single sectors which consist of only one node. Because these single sectors have no merits for grouping, we have to eliminate them or incorporate them into other sectors. In our scheme, a single sector joins one of adjacent sectors. After the sector determination, sector managers register themselves into the sink. To reduce inter-sector interference, each sector communicates with the sink using direct-sequence spread spectrum (DSSS). Each sector employs a unique spreading code. All sensors in a sector transmit a message using the spreading code and the code is assigned when the sector manager registers itself into the sink. For instance, the first sector manager to register is assigned the first code on a predefined list, the second sector manager to register is assigned the second code, and so on. Then sensors establish pairwise keys with other sensors in the same sector. The membership and the structure of sectors highly depend on which CH election scheme is used for the sector formation. Besides, it also affects the probability of success of pairwise key establishments in a sector. We deal with this effect in Section 4.3

### 4.3. Pairwise Key Estalbishments in Sectors

In a sector, a sensor may recognize some sensors which do not share common assigned keys. These sensors are referred to as insecure sensors in this paper. A sensor can indirectly establish pairwise keys with the insecure sensors using the helper nodes. However, if all sensors in a sector perform this indirect pairwise key establishment individually, it causes a lot of communication and computation overhead. To reduce this overhead, we use the sector manager. The reason why we use the sector manager is that it is directly connected to the most of its members. First, the sector manager establishes pairwise keys with its insecure members using the helper nodes. If all helper nodes do not share a common key with an insecure sensor, the sector manager establishes pairwise keys with the insecure nodes via the help of the sink. This is because the sink has a key pool of all keys assigned to sensors in the network. In fact, this key establishment using the sink causes a lot of communication overhead. This is because the distance between a sector manager and the sink is fairly long in most cases. Then the sector manager broadcasts the list of members, and each member establishes pairwise keys with its insecure members via the help of its sector manager. That is, a sector manager which is requested to distribute a pairwise key by a member generates a pairwise key and distributes it to two members. Therefore, the success probability of intra-sector key establishments is highly depends on the success probability of key establishments between the sector manager and its memebers. So, we need to analyze the success probability of key establishments between a sector manager and its members by varying the key assignment probability and the sector formation scheme.

The number of helper nodes has a greatest impact on the success of pairwise key establishment between a sector manager and its members. The number of helper nodes highly depends on the probability that a key is assigned to a sensor from the key pool. That is, if this probability is high, the number of helper nodes increases and the success probability of intra-sector key establishments increases. Second, a cluster formation scheme used for sector determination makes a difference in membership, size, and structure of sectors. For instance, some sector managers can have many helper nodes. We performed a simulation to verify the above descriptions. In the simulation, 100 sensors which have 50 keys were randomly deployed in a simulation area of 100 m × 100 m, and each sector manager tried to establish pairwise keys with its members. We varied the size of key pool to make a difference in the key distribution probability and made sectors using four different cluster formation schemes (that is, LIDCP [7], HCCP [7], Two Phase [16], and VCA [9])

**Figure 2.**
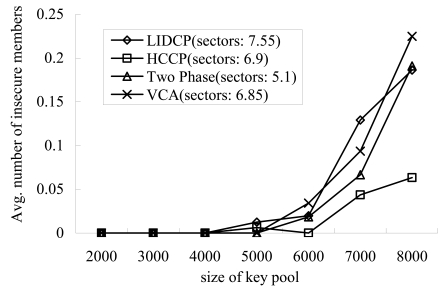
Insecure members vs. size of key pool.

Figure 2 shows the variation of member nodes with which a sector manager could not establish pairwise keys using helper nodes. Figure 2 also shows the difference between four cluster formation schemes. As shown in Figure 2, all schemes increase the number of failure nodes as the size of key pool increases. This is because the probability that a key exists in a sensor decreases and consequently makes the reduction in the number of helper nodes. Nevertheless, HCCP significantly reduces the failure nodes.

**Figure 3.**
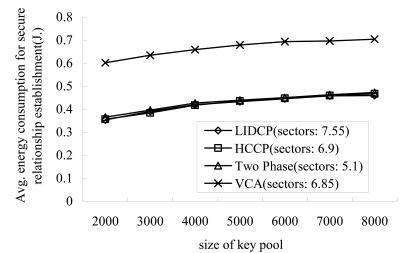
Energy consumption for intra-sector key establishments vs. size of key pool.

Figure 3 shows the variation of energy consumption for intra-sector key establishments as the size of key pool increases. Here, the intra-sector key establishment includes the initial sector formation, key establishments between sector managers and their members. As shown in Figure 3, VCA consumes more energy than the three other schemes because it makes all sensors exchange three messages for sector formation. The other three schemes consume almost same amount of energy for the intra-sector key establishments. Therefore, in this paper we decided to make sectors using HCCP.

## 5. Secure Cluster Head Election

After the pairwise keys between sensors in a sector are established, sensors should elect a node which plays the role of CH in this round. For simplicity, we assume that no collision occured in the MAC layers of sensors during the CH election. This assumption can be actualized using a broadcast order which is predetermined for broadcast of fulfillments in each sector.

As shown in Section 3, a CH election scheme using a sum of random values has some problems. They enable the manipulation attack by a last node which is expected to transmit the fulfillment value. Our scheme adopts the following strategies to prevent the manipulation attack.

1. Initially, the transmission order of fulfillment values is scheduled by the order of IDs.
2. Sensors which do not follow the order are scheduled to transmit its fulfillment value before anyone else. Thanks to the synchronization phase, every sensor can transmit its fulfillment value without interference even though the transmission order is changed.
3. If a sensor does not follow the schedule more than one time, it is excluded from the sector member list by other sensors.

**Figure 4.**
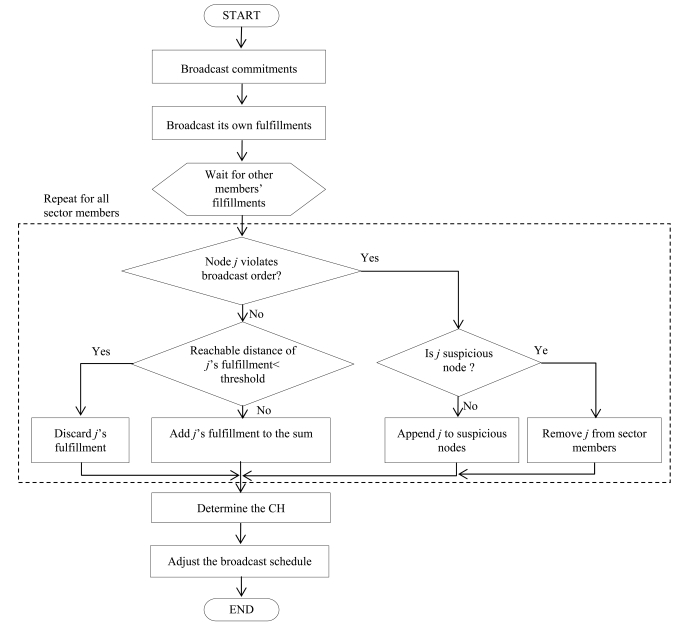
Flow chart of secure cluster head election using signal strength estimation and ordered transmission.

Besides, attackers in a CH election scheme using a sum of random values can collapse a CH agreement by lowering the transmission power when they transmit their fulfillment values. To defeat this agreement prevention attack, our scheme employs the received signal strength. Figure 4 shows the flowchart of the proposed cluster head election using the signal strength estimation and the ordered transmissions.

### 5.1. Commitment Broadcast

After determining the sector, each sensor synchronizes its clock with other members in its sector. Then, each sensor sets its timer interval to a predefined value. The timer interval is long enough to accommodate all later steps as well as data transmissions. Then each sensor generates its random number and encrypts it with pairwise keys shared with other sensors to make commitments. The commitments are generated as many as the number of other sensors. Each sensor makes a list of the commitments in the order of IDs and broadcasts the list. After initial sector formation, distance between any two sensors in a sector two hops and it is extended to at most four hops after join of single sectors. Therefore, each sensor broadcasts the list with the power with which a message can reach four hops away nodes. Sensors receiving the list first check whether the sender is a member in their sectors or not. If the sender is not the member, the receivers discard the message. Otherwise, the receivers pick up its commitment from the list and decrypt it to store with the sender's ID.

### 5.2. Broadcast of Fulfillment Value

The commitments broadcasted by sensors can take part in the generation of the sum of random values only if corresponding fulfillment values are received from the sensors. In this step, each sensor broadcasts the random number which was used for commitment generation with the transmission power with which a message can reach four hops away nodes. Each sensor knows the broadcast order of fulfillment values (random numbers) and should follow the order. After the sector formation, this order is configured with an ascending order of IDs of sector members. If a sensor violates this order, the sensor is identified as a suspicious node and recorded in the suspicious node list. Besides, the message from a suspicious node is discarded. If a sensor broadcasts the fulfillment value in its correct order, receiving sensors compare it with the corresponding commitment to check the equality. If they are equal, the receivers store the sender into normal node list. If a suspicious node violates the broadcast order again, the receivers exclude it from the member list and the suspicious node list. Therefore, a compromised node can prevent a specific node from being a CH only once by suppressing its fulfillment value transmission. Afterward, it is forced to broadcast its fulfillment value in the first order. If it tries to suppress its fulfillment transmission again or delays its transmission, other legitimate sensors eliminate it from the member list as well as discard the fulfillment value.

Although a sensor transmits its fulfillment value, some sensors cannot receive it when the transmission power of the message is lower than a specific level. That is, we infer a node as an attacker trying to impair the agreement property if its message cannot reach at most four hop nodes. Receivers can recognize this trial using the received signal strength. However, received signal strength can be different at each sensor due to an obstacle or a propagation error. To deal with this problem, we set a specific level of signal strength to a threshold. The distance of the threshold ranges from three hops to four hops and it is close to four hops. Even though this technique cannot avoid the agreement prevention attack perfectly, the benefits of attackers are significantly reduced. Note that attackers should transmit a message with a power level which reaches over three hop away nodes. Therefore, the number of split clusters is reduced. If a message can propagate over the threshold, the receivers keep the message. Otherwise, it discards the message. We assume that the energy model in [1] is employed in the energy consumption of transmitters and receivers. Assuming the two-ray ground reflection model is used for radio propagation, a receiving node can calculate the transmission power of a sender transmitting a fulfillment value(*P_t_*) by Equation (1):

(1) $$P_{t}=\frac{P_{r}d^{4}L}{G_{t}G_{r}h_{t}^{2}h_{r}^{2}}$$

where, *P_r_* is the received power, *d* is the Euclidean distance, and *L* is the system loss. Besides, *G_t_* and *G_r_* are antenna gains and *h_t_* and *h_r_* are antenna heights. If a receiving node can know the transmission power of a sender, it can estimate the maximum reachable distance by the power (*d_r_*) by Equation (2):

(2) $$d_{r}=\sqrt{\sqrt{\frac{P_{t}}{E_{\text{two}\_\text{ray}\_\text{amp}}\times b}}}$$

Here, *E_two_ray_amp_* is the energy consumed by the amplifier and *b* is the bandwidth of the channel. If *d_r_* is smaller than a predetermined threshold, the receivers discard the received fulfillment value. This rejection of fulfillment value based on the received signal strength mitigates the impairment of the agreement property. That is, this mechanism alleviates the splitting of a cluster.

### 5.3. Random Value Generation and CH Election

If all random numbers are gathered from other sensors, each sensor generates a sum of the random numbers. Then they divide the sum by the number of normal nodes to get the remainder. Note that all sensors keep the list of normal nodes which follow the broadcast order correctly. The remainder is the index of a node which is elected as CH in the normal node list.

Each sensor finds out the sensors which do not transmit their fulfillment value. This non-transmission of fulfillment value may be resulted from an attacker which prevents a sensor from being a CH or a message loss. Therefore, each sensor inserts these nodes into the suspicious node list at first. If these nodes repeat the same misbehavior, each sensor erases these nodes from the member list and the suspicious node list.

### 5.4. Adjustment of Broadcast Order

Now it is time for each sensor to readjust the broadcast order of fulfillments. In the broadcast order, the suspicious nodes are moved to the first places and normal nodes follow them. Namely, the broadcast order of the next round is generated by concatenating the suspicious node list and the normal node list.

The elected CH in each sector generates a TDMA schedule and broadcasts it. All members compute their transmission time and rest time in line with this schedule. They transmit their sensed data to the CH in their allowed time slots, and the CH transmits the aggregated data to the sink. This procedure is repeated until the timer which was set in the commitment broadcast step expires. If the timer expires, each sensor restarts the commitment broadcast step in Section 5.1.

## 6. Simulations

We built the simulation environment to evaluate our scheme in terms of security and efficiency (especially, energy efficiency). In the simulation environment, 100 nodes were randomly deployed in a 100 m × 100 m area, and the sink was at (50 m, 175 m) position. The energy model employed in the simulation adopted that of [1]. To concentrate on the evaluation of security and energy efficiency for the CH election, we did not implement the data transmissions (sensor-CH and CH-sink). We ran each scheme 20 times for each number of compromised nodes, and the network topology and the compromised nodes were changed in each run. In a sector, all compromised nodes invoke the same kind of attack. This is because two objectives of compromised nodes (that is, impairment of non-manipulability and impairment of agreement) are conflict with each other. To manipulate the CH election result in a sector, all compromised nodes in the sector cooperate with each other. That is, only one compromised node does not send its fulfillment value while others behave like legal nodes. If the non-sender is excluded from its sector, one node among other compromised nodes repeats the same misbehavior. If the number of compromised nodes increases, they have more chances to become a CH because most of them normally act. Simulation results were collected to compute statistical representatives. All these representatives have 95% confidence intervals. Table 1 shows the parameters and their values employed in the simulations.

We compared our scheme with the commitment based scheme and the seed based scheme. This is because our scheme is also a kind of random value based schemes like the commitment based scheme and the seed based scheme. Although many other schemes have been proposed so far, their CH election method is greatly different from that of random valued based schemes. Their CH election highly depends on a weight value which can be deceitfully claimed by an attacker. However, random value based schemes elect a CH in a random manner to avoid the intentional cheat. So, comparing our scheme with them is unfair. To facilitate the security comparison of our scheme and other schemes, we developed the following metrics.

- Average number of CHs generated during the CH election process: This value is the metric for evaluating the agreement property. If this value is large, it is evidence that the agreement property is severely impaired.
- Frequency of the case in which compromised nodes are elected as CHs: This value is the metric for evaluating the non-manipulability. A CH election scheme should keep this value low.
- Frequency of the case in which CH election result is changed: This value is the metric for evaluating the non-manipulability. If this metric and the previous metric increase, it is evidence that the CH election scheme cannot prevent the attackers from suppressing the transmission of fulfillment values.

Besides, we developed the following metric to compare our scheme with other schemes in terms of energy efficiency.

- Total energy consumption: This value is the sum of energy consumed for sector formation, pairwise key establishments, and periodic CH elections at all sensors. If this value is large, it is evidence that the energy efficiency of the CH election scheme is low.

### 6.1. Security Evaluation

**Figure 5.**
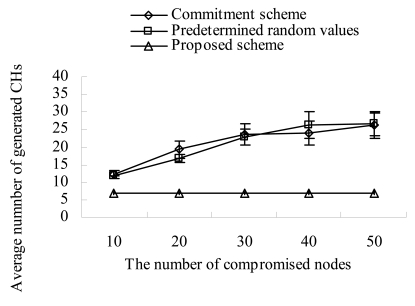
Generated CHs vs. compromised nodes.

Figure 5 shows the variation of average number of CHs with the increase of compromised nodes. As shown in Figure 5, the commitment based scheme and the seed based scheme (depicted as predetermined random values) are very vulnerable to the agreement impairment attack. In those schemes, even though a compromised node broadcasts a fulfillment value with a low transmission power, sensors do not check the received signal strength of the message.

**Figure 6.**
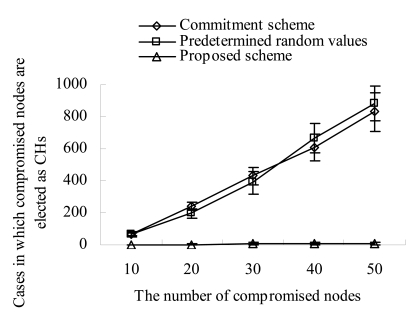
Cases in which compromised nodes are elected as CHs vs. compromised nodes.

Therefore, some receivers in the same sector have a different sum of random numbers and this makes them elect a different node as their CH, and the number of CHs in the sector increases. As compromised nodes proliferate, their fulfillment broadcasted with a low transmission power makes more different sums of random numbers in a sector. This creates a number of CHs and weakens the advantage of cluster structure. On the contrary, in our scheme, receivers measure the transmission power of a fulfillment value and estimate the corresponding reachable distance. If a fulfillment value cannot reach a specified threshold, they discard the value. That is, because our scheme blocks the agreement impairment attacks, it produces almost same number of clusters (CHs) as the number of sectors (that is, on average 6.9 sectors). Besides, it produces almost constant number of clusters regardless of the increase of compromised nodes. Figure 6 shows the cases in which the compromised nodes are elected as CHs as the number of compromised nodes increases. In the commitment based scheme and the seed based scheme, the compromised nodes can continuously prevent the legitimate nodes from being elected as CHs by suppressing their transmissions of fulfillment values. This continuous prevention gives the chances of being CHs to the compromised nodes. Therefore, as the number of compromised nodes increases, they can get more chances of being CHs. In our scheme, a compromised node can get the chance of being a CH only once. This is because it would be excluded if it performs the malicious action two times. As a result, the cases in which the compromised nodes are elected as CHs decrease remarkably in our scheme.

**Figure 7.**
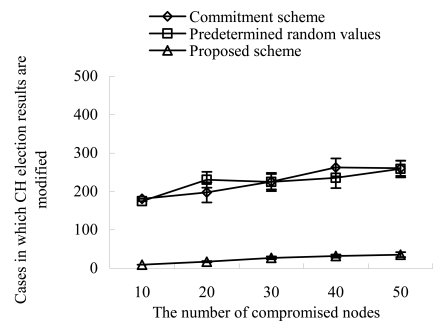
Cases in which CH election results are modified vs. compromised nodes.

Figure 7 shows the cases in which the CH election results are changed as the number of compromised nodes increases. In the commitment based scheme and the seed based scheme, the compromised nodes can change the CH election result by suppressing the transmission of their fulfillment values. However, the increase in compromised nodes does not always cause the increase in the changes of CH election results. This is because no matter how many compromised nodes exist in a sector, only the last sender of fulfillment values can change the CH election result. Our scheme greatly reduces the changes as compared with other schemes. This is because the compromised nodes can take only one chance to change the CH election result by suppressing their fulfillment value. If they take the malicious action two times, they are excluded from the sector by other legitimate nodes.

### 6.2. Energy Efficiency Evaluation

**Figure 8.**
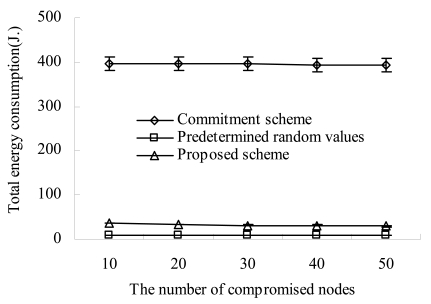
Total energy consumption vs. compromised nodes.

Figure 8 shows the total amount of energy consumption during the simulation as the number of compromised nodes increases. Because all schemes employ the same sector formation protocol (that is, HCCP), the amounts of energy consumed for sector formation and key establishments within sectors are equal. However, because they employ a different method to transmit the commitment and the fulfillment value, their energy consumption for the CH election is different from each other.

As shown in Figure 8, our scheme consumes more amount of energy than the seed based scheme. This is because our scheme makes each sensor transmit its commitment every CH election round while the seed based scheme makes each sensor transmit its seed (that is, commitment) just once. Even though the seed based scheme reduces the energy consumption by avoiding periodic transmission of commitment, attackers can predict the results of CH elections. This predictability makes a lot of attacks available [10]. In our scheme, because sensors transmit their commitment every CH election round and they are encrypted with pairwise keys, attackers cannot predict which nodes are elected as CHs. Besides, our scheme employs sensors' energy more efficiently than the commitment based scheme. This is because the commitment based scheme makes a sensor transmit its commitment to other sensors in a peer-to-peer manner.

## 7. Synchronization Issue

Our scheme satisfies most of the required properties defined in [10]. First, our scheme satisfies the strong non-manipulability that the commitment based scheme and the seed based scheme do not satisfy. That is, our scheme prevents an attacker from manipulating the CH election result by excluding it from the member list. Second, although a sector is partitioned into many clusters in a round, they can be incorporated into one cluster again if sensors follow the broadcast order of fulfillment values. So, our scheme is adaptive. Third, the CH election in our scheme does not rely on a specific node, so our scheme tolerates the failure of a node. Lastly, even though our scheme is worse than the seed based scheme in terms of energy efficiency, it provides a desirable property of a CH election scheme (that is, unpredictability).

Generally, a periodic CH election in a network requires the time synchronization between sensors. The weight based schemes require the global synchronization between sensors. This is because a CH election in a region is affected by CH elections of neighboring other nodes. However, our scheme does not need the global synchronization. This is because a CH election in a sector does not affect that of other sectors. Therefore, the synchronization in a sector is only required in our scheme.

A lot of schemes for local and global network synchronization have been proposed so far. Recently, TinySerSync [17] which was proposed by Sun *et al.* presents a local synchronization scheme between neighbors which share a pairwise key and a global synchronization scheme using μTESLA. Because sensors in a sector share a pairwise key with each other in our scheme, they can synchronize with each other using the local synchronization scheme of TinySerSync.

## 8. Conclusions

In this paper, we have shown that the existing CH election schemes did not deal effectively with some malicious actions of smart attackers. We first identified those malicious actions and proposed two mechanisms to provide the resiliency against them, namely the preservation of order for fulfillment value broadcast and the message abolition prevention using received signal strength. Our simulation results showed that these two mechanisms prevent the malicious actions of smart attackers with a little drop in energy efficiency.

For future work, we are going to study the performance variance of our scheme under the environment where sensors are mobile. For long lived networks, we need to deal with join of new nodes during network operation. For that purpose, we need a scheme which concerns inter-generation pairwise keys establishments. Besides, we are planning to incorporate our scheme with the cluster based key management to design a new key management scheme which is based on the secure CH election.

## Figures and Tables

**Table 1.** Simulation parameters.

**Parameter**	**Value**
Simulation time	3,600 sec.
Initial energy	10 Joules/battery
Bandwidth	1 Mbps
Data packet size	500 bytes
Packet header size	25 bytes
Number of compromised nodes	10 ∼ 50
Compromise time distribution	Random, 3 ∼ 1,800 sec.
Neighbor radius	30 meters
Clustering protocol for sector formation	HCCP [8]
Expiration time of CH election timer	20 sec.

## References

[1] Heinzelman W., Chandrakasan A.P., Balakrishnan H. (2002). An application-specific protocol architecture for wireless microsensor networks. IEEE Trans. Wireless Commun..

[2] Younis O., Fahmy S. (2004). HEED: a hybrid, energy-efficient, distributed clustering approach for ad hoc sensor networks. IEEE Trans. Mobile Comput..

[3] Gupta G., Younis M. (2003). Performance evaluation of load-balanced clustering of wireless sensor networks.

[4] Jolly G., Kuscu M.C., Kokate P., Younis M. A low-energy key management protocol for wireless sensor networks.

[5] Eltoweissy M., Moharrum M., Mukkamala R. (2006). Dynamic key management in sensor networks. IEEE Commun. Mag..

[6] Chatterjee M., Das S., Turgut D. An on-demand weighted clustering algorithm (WCA) for ad hoc networks.

[7] Gerla M., Chiang C. (1995). Multicluster, mobile, multimedia radio network. ACM-Baltzer J. Wireless Netw..

[8] Basu P., Khan N., Little T.D.C. A mobility based metric for clustering in mobile ad hoc networks.

[9] Qin M., Zimmermann R. An energy-efficient voting-based clustering algorithm for sensor networks.

[10] Sirivianos M, Westhoff M., Armknecht F., Girao J. Non-manipulable aggregator node election protocols for wireless sensor networks.

[11] Eschenauer L., Gilgor V.D. A key management scheme for distributed sensor networks.

[12] Chan H., Perrig A., Song D. Random key predistribution schemes for distributed sensor networks.

[13] Ferreira A.C., Vilaca M.A., Oliveira L.B., Habib E., Wong H.C., Loureriro A.A. On the security of cluster-based communication protocols for wireless sensor networks.

[14] Perrig A., Szewczyk R., Wen V., Culler D., Tygar J.D. (2002). SPINS: Security protocols for sensor networks. Wireless Netw..

[15] Oliveira L.B., Wong H.C., Bern M., Dahab R., Loureiro A.A.F. SecLEACH - A random key distribution solution for securing clustered sensor networks.

[16] Wang K., Cho G. Two phases based cluster formation scheme for mobile ad hoc networks.

[17] Sun K., Ning R., Wang C., Liu A., Zhou Y. TinySeRSync: secure and resilient time synchronization in wireless sensor networks.

